# A bioactive cell sheet interface regulates scaffold–host interactions for cartilage regeneration

**DOI:** 10.1002/btm2.70177

**Published:** 2026-09-22

**Authors:** Yi Chieh Chang, Cherng‐Shyang Chang, Chung‐Kan Tsao

**Affiliations:** ^1^ International Master Science Program in Reconstructive Microsurgery College of Medicine, Chang Gung University Taoyuan Taiwan; ^2^ Center for Tissue Engineering Chang Gung Memorial Hospital at Linkou Taoyuan Taiwan; ^3^ Division of Reconstructive Microsurgery, Department of Plastic and Reconstructive Surgery Chang Gung Memorial Hospital at Linkou Taoyuan Taiwan

**Keywords:** cartilage tissue engineering, cell sheet engineering, encapsulation strategy, host integration, implantation environment, scaffold–host interaction, vascularization

## Abstract

Cartilage tissue engineering remains challenged by uncontrolled scaffold–host interactions after implantation, particularly host tissue infiltration that can disrupt cartilage formation and compromise scaffold integrity. Although biomaterial‐based encapsulation strategies have been developed to reduce host interference, excessive restriction of scaffold–host interface may also limit the exchange of nutrients, oxygen, and metabolic wastes. Therefore, an effective encapsulation strategy must protect the developing tissue from excessive host infiltration while maintaining a permissive environment for tissue formation. Here, we engineered a fibroblast‐based cell sheet to regulate scaffold–host interactions in a nude mice implantation model. Fibroblast cell sheet wrapping protected the scaffold by limiting host cellular infiltration, but cartilage extracellular matrix (ECM) formation was also reduced within the scaffold. Notably, EPC incorporation into the cell sheet restored cartilage ECM deposition and enhanced peri‐scaffold vascularization. These results demonstrate that cell sheet‐based encapsulation can provide not only a protective function against excessive host infiltration but also a bioactive interface that regulates scaffold–host interactions, with EPC incorporation promoting peri‐scaffold vascularization and supporting cartilage matrix formation within the scaffold.

**Translation Impact Statement**

Uncontrolled host tissue infiltration is a common challenge for tissue‐engineered cartilage scaffolds following implantation and can compromise the structural stability and regenerative performance. In this study, we show that fibroblast‐based cell sheet encapsulation can limit host cellular infiltration and preserve scaffold integrity, but also restrict cartilage matrix formation. Incorporation of endothelial progenitor cells into the cell sheet enhanced peri‐scaffold vascularization and restored cartilage extracellular matrix deposition. These results suggest that this engineered cell sheet functions not only as a protective interface but also as a bioactive interface that modulates the scaffold–host interactions and supports cartilage regeneration. This approach highlights the potential of cell sheet‐based encapsulation to provide scaffold protection while actively regulating the scaffold–host interactions through a bioactive interface.

## 1 INTRODUCTION

Cell‐based tissue engineering techniques have achieved significant progress in cartilage regeneration. However, their regenerative performance remains highly dependent on the implantation environment. After implantation, scaffolds inevitably interact with host tissue, triggering host responses, including tissue infiltration, neovascularization ingrowth, and immune cell recruitment.^1^, ^2^, ^3^ Although these processes are essential for scaffold integration, excessive or uncontrolled host responses can interfere with regenerative outcomes by occupying scaffold inner space, impeding cartilage extracellular matrix (ECM) formation, damaging scaffold structure, and ultimately leading to scaffold failure.^1^, ^4^ Therefore, balancing scaffold protection with host tissue integration remains a main challenge in cartilage tissue engineering.

In articular cartilage repair, cartilage scaffolds implanted in joint defects support stable cartilage regeneration, largely due to the regular mechanical loading and the relatively homogeneous and avascular environment.^5^, ^6^ However, in non‐joint regions such as tracheal repair and craniofacial reconstruction (e.g., microtia), implanted cartilage scaffolds are exposed to heterogeneous environments characterized by abundant connective tissues, immune activation, extensive vascularization, and dynamic mechanical forces.^7^, ^8^, ^9^ Accumulating evidence suggests that excessive vascular ingrowth within cartilage tissue can disrupt cartilage homeostasis.^5^, ^10^, ^11^ Therefore, limiting uncontrolled host responses has been considered as a critical challenge for maintaining chondrogenic performance. To address this issue, scaffold encapsulation strategies have been proposed. Previous studies have shown that encapsulating scaffolds with synthetic polymer membranes or hydrogels can reduce host response, establish immunoisolation, and enhance cell survival.^12^, ^13^, ^14^, ^15^, ^16^ These studies reveal that a physical barrier can suppress host responses and stabilize scaffolds within the implanted environment.

In addition to scaffold encapsulation, scaffold vascularization is also crucial to support nutrient delivery, oxygen supply, and metabolic needs after implantation.^17^, ^18^ In tracheal tissue engineering, early phase vascularization is required to support graft survival and host integration.^19^, ^20^ Previous studies showed that using heterotopic implantation of tracheal scaffolds in limb muscle or an abdominal muscle flap for pre‐vascularization can improve cartilage scaffold performance after transplantation.^19^, ^20^ Notably, pre‐vascularization mainly originates from surrounding muscle, suggesting the advantage of peri‐scaffold vascularization in supporting tracheal scaffolds. However, achieving spatial control of vascularization remains a significant challenge, as it requires promoting peri‐scaffold vascularization while preventing excessive vascular ingrowth simultaneously.

However, scaffold design alone may not be sufficient to control host response after implantation, particularly host tissue infiltration and vascularization. Thus, we sought to develop a bioengineered interface at scaffold–host boundary that complements scaffold design by providing scaffold protection and regulating scaffold–host interaction. It is reminiscent of the native tracheal structure, in which each cartilaginous ring is enclosed by intercartilaginous ligaments and surrounded by an adventitial layer that forms as a fibrovascular compartment.^7^, ^21^ We hypothesized that this fibrovascular compartment functions as a regulatory interface that not only prevents surrounding tissue infiltration but also spatially regulates vascular growth. Specifically, this fibrovascular compartment is primarily composed of fibroblasts.^7^ Inspired by the native structure, we sought to recapitulate the fibrovascular compartment around the cartilage scaffold using a fibroblast‐based cell sheet.

We fabricated a fibroblast‐based cell sheet (FB cell sheet) to encapsulate the cartilage scaffold. Using a subcutaneous implantation model, we first evaluated its protective effect on host tissue infiltration using cell‐free scaffolds with or without FB cell sheet wrapping (CF and CF‐FB). Next, we applied the cell sheet to chondrocyte‐laden scaffolds. To introduce vascular potential into the cell sheet, endothelial progenitor cells (EPCs) were incorporated into the FB cell sheet to generate FB/EPC cell sheets. We compared non‐encapsulated scaffolds (CH), FB cell sheet encapsulated scaffolds (CH‐FB), and FB/EPC cell sheet encapsulated scaffolds (CH‐FB/EPC) to determine how cell sheet encapsulation affects cartilage regeneration and vascularization at the scaffold–host interface in a heterogeneous implantation environment. We found that fibroblast‐based cell sheet encapsulation limited host cellular infiltration and preserved scaffold integrity, but also restricted cartilage matrix formation. Incorporation of EPCs into the cell sheet restored cartilage ECM deposition and enhanced peri‐scaffold vascularization. These results suggest that the engineered cell sheet provides a complementary approach to scaffold design by protecting the scaffold from host tissue infiltration while functioning as a bioactive interface that modulates vascularization and supports cartilage regeneration.

## 2 RESULTS

### 2.1 Construction and spatial characterization of EPC‐incorporated fibroblast cell sheet

To fabricate a dense and cohesive cell sheet, we adopted a layer‐by‐layer (LbL) assembly approach to fabricate the cell sheet.^22^, ^23^, ^24^ As shown in Figure 1a, the cells were repeatedly coated during each time of cell seeding. Accordingly, the coated cells were seeded onto the culture dish daily, resulting in bottom‐up accumulation (left panel in Figure 1b).^23^, ^24^ After that, a dense and cohesive cell sheet could be detached from the culture dish as an intact sheet (right image in Figure 1b).

**FIGURE 1:**
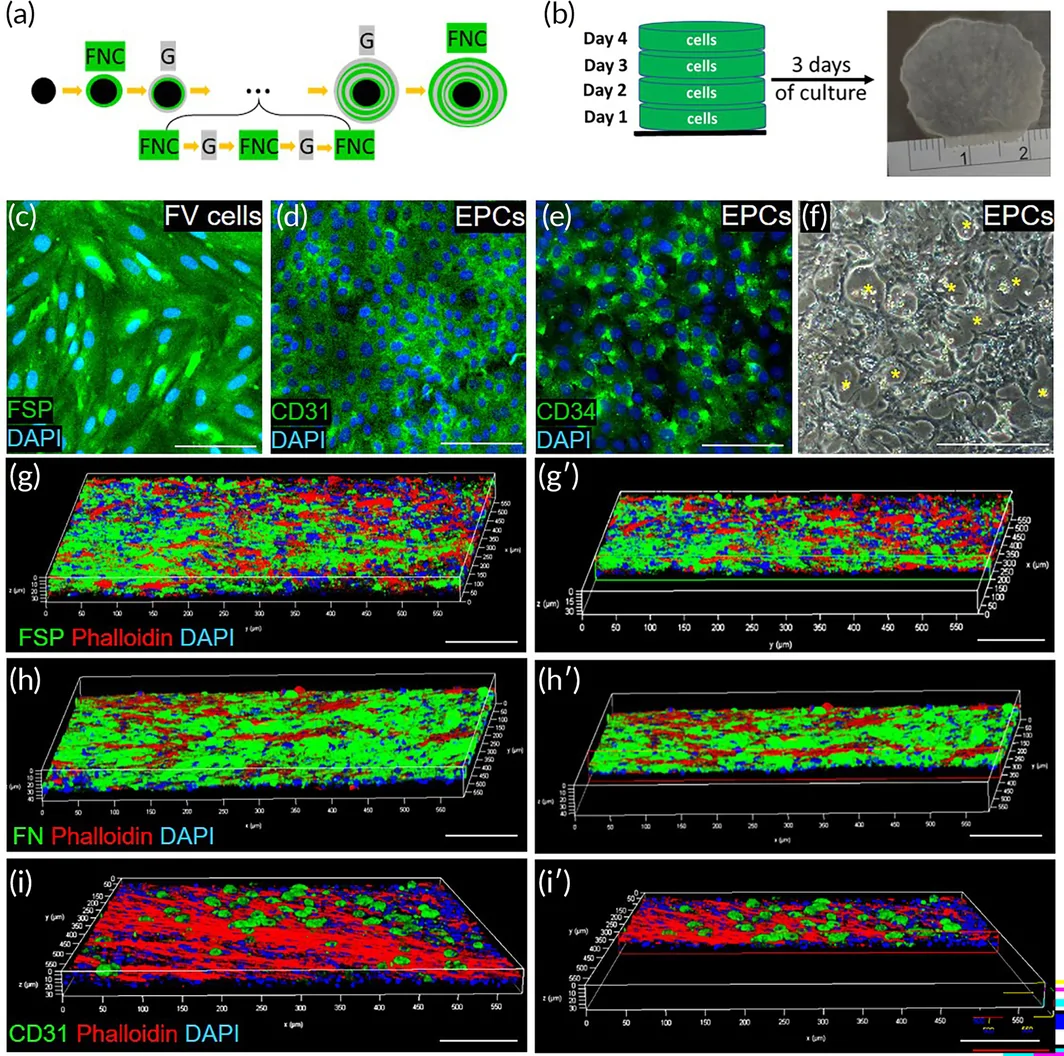
Construction and spatial characterization of EPC‐incorporated fibroblast cell sheet. (a) Schematic illustration of the layer‐by‐layer (LbL) assembly approach for cell sheet fabrication. Cells were sequentially coated with fibronectin coating mix (FNC) and gelatin (G) for four deposition cycles, followed by an additional FNC coating prior to cell seeding. (b) The coated cells were seeded daily for four consecutive days and followed by 3 days of culture. The mature cell sheet could be completely detached from the culture dish. (c) Cells isolated from tracheal fibrovascular (FV) compartment. At passage 3 of primary culture, these cells expressed fibroblast surface protein (FSP). Scale bar = 100 μm. (d, e) Cells were isolated from adipose‐derived stromal vascular fraction and subjected to selective primary culture. At passage 3, these selected cells expressed the markers CD31 (d) and CD34 (e), which are markers of endothelial cells and progenitor cells, respectively. Scale bar = 100 μm. (f) Tube formation assay showing capillary‐like structure (asterisks). Scale bar = 500 μm. (g–i′) 3D confocal reconstructions of the fibroblast cell sheet stained for fibroblast surface protein (FSP) (g), fibronectin (FN) (h), and CD31 (i), with corresponding cross‐sectional views (g′–i′). FN and fibroblast surface protein were broadly distributed throughout the sheet (g′,h′), whereas CD31‐positive EPCs were sparsely detected and primarily localized to the top layer (i′). Scale bar = 100 μm.

To isolate cells from the tracheal fibrovascular compartment, with minimizing contamination by chondrocytes, a shortened enzymatic digestion protocol was used. At passage 3, the isolated cells predominantly exhibited a fibroblast phenotype, as indicated by fibroblast surface protein (FSP) expression (Figure 1c). The lack of vascular‐associated cells in the primary culture suggested the need to incorporate an additional vascular component into the fibroblast‐based cell sheet. Therefore, endothelial progenitor cells (EPCs) were isolated from white adipose tissue as previously described.^25^ At passage 3 of primary culture, these cells expressed CD31 and formed vascular tube‐like structure in tube formation assay, indicating endothelial identity and functionality, respectively (Figure 1d,f). Further, the presence of CD34 suggests a progenitor‐like phenotype consistent with EPCs (Figure 1e). Together, these findings confirmed that these isolated cells possess EPC characteristics.

The fibroblast‐based cell sheet (FB cell sheet) was fabricated using the LbL assembly approach described above. EPC‐incorporated cell sheet (FB/EPC cell sheet) was generated by incorporating EPCs into the top layer of the FB cell sheet. Immunofluorescent staining with 3D confocal reconstruction revealed the spatial organization of the FB/EPC sheet. Fibroblast surface protein (FSP) and fibronectin (FN) were broadly distributed throughout the cell sheet as confirmed by cross‐sectional views (Figure 1g,h′). Whereas CD31‐positive EPCs were detected in a subset of cells and were mainly localized to the top layer (Figure 1i,i′). These results indicate a spatial organization of the cell sheet, in which fibroblasts were predominantly distributed throughout the bulk, and EPCs were localized to the top layer.

### 2.2 PCL scaffolds provide a chondrogenic platform for evaluating fibroblast‐based encapsulation

To investigate the effects of cell sheet, a polycaprolactone (PCL) scaffold was used as a testing platform. PCL is a conventional and widely used biomaterial in tissue engineering and has been reported to permit infiltration of surrounding cells after implantation,^20^ making it suitable for evaluating the effects of scaffold encapsulation. Here, PCL was fabricated as a planar porous scaffold with a pore diameter of approximately 0.3 mm (Figure 2a). Chondrocytes were seeded onto the scaffold and assessed for scaffold chondrogenesis before implantation. At day 3 post‐seeding, widespread SOX9 expression confirmed the chondrogenic identity of the cells on the scaffold (Figure 2b). By day 14, most cells expressed aggrecan (ACN) and type II collagen (Col II) (Figure 2c,d), indicating the initiation of cartilage ECM accumulation within the scaffold.

**FIGURE 2:**
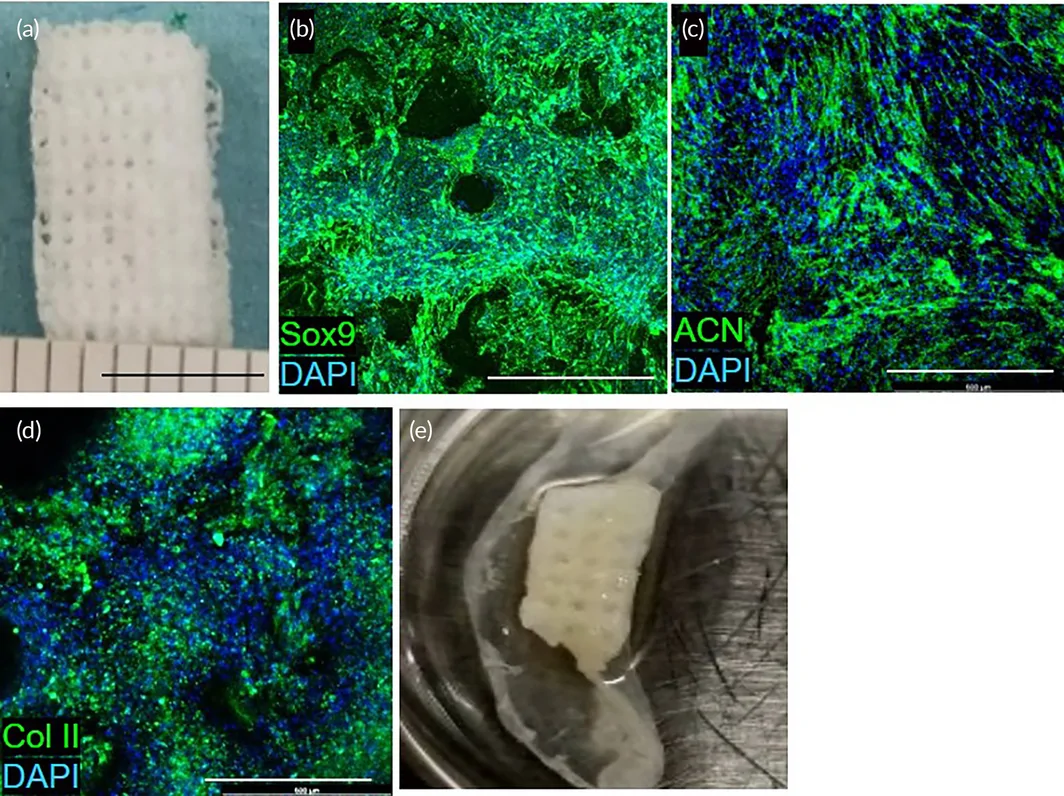
PCL scaffolds provide a chondrogenic platform for evaluating fibroblast‐based encapsulation. (a) Representative images of salt‐leached PCL patch scaffolds with macropores organized throughout the scaffold. Grid spacing, 1 mm. Scale bar = 5 mm. (b) Sox9 staining at day 3 post‐seeding showed widespread nuclear expression in chondrocytes within the scaffold. Scale bar = 500 μm. (c) At day 14, aggrecan (ACN) expression was detected in most chondrocytes. Scale bar = 500 μm. (d) Type II collagen (Col II) was prominently expressed intracellularly in chondrocytes on the scaffold. Scale bar = 500 μm. (e) A planar scaffold was wrapped with a fibroblast cell sheet.

A cell sheet cultured in a 3.5‐cm culture dish was sufficient to completely encapsulate the scaffold (Figure 2e), with the fibroblast layer facing the scaffold, and the EPC‐containing layer facing outwards. The cell sheet wrapped scaffolds were immediately implanted into the subcutaneous region in nude mice.

### 2.3 Fibroblast cell sheet encapsulation limits host cell infiltration

To determine whether fibroblast cell sheet encapsulation could alter host–scaffold interactions, cell free (CF) PCL scaffolds with or without FB cell sheet wrapping were implanted subcutaneously in nude mice. After 2 weeks, CF scaffolds showed extensive host cell infiltration, as evidenced by H&E and DAPI staining (Figure 3a,d). Whereas scaffolds wrapped with a FB cell sheet (CF‐FB) exhibited markedly reduced host cell infiltration (Figure 3b,e). Quantitative analysis supported these observations that both hematoxylin‐positive area and DAPI‐positive area were significantly lower in CF‐FB scaffolds than in CF scaffolds (Figure 3c,f). Moreover, partial degradation of scaffold structure was observed in CF scaffolds (arrow in Figure 3a), whereas the structural integrity of CF‐FB scaffolds was largely preserved (Figure 3b). These results indicate that FB cell sheet wrapping attenuates host cellular infiltration while maintaining the structural integrity of the implanted scaffolds.

**FIGURE 3:**
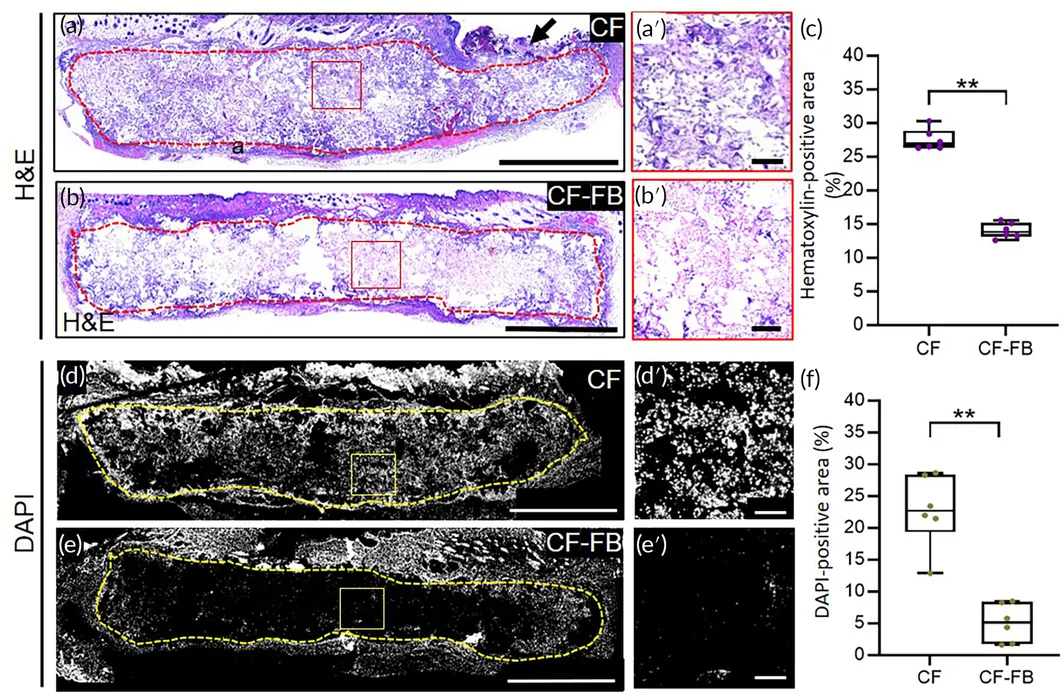
Fibroblast cell sheet encapsulation limits host cell infiltration. Cell‐free scaffolds with or without fibroblast cell sheet wrapping were implanted subcutaneously into nude mice and harvested after 14 days. CF denoted unwrapped cell‐free scaffolds, while CF‐FB indicated scaffolds wrapped with a fibroblast cell sheet. Cross views of explanted scaffolds were shown in the left panels (scale bar = 2 mm in (a), (b), (d), and (e)), with boxed regions magnified in right images (scale bar = 200 μm in (a′), (b′), (d′), and (e′)). (a–c) H&E staining showed abundant host cell infiltration into the interior of CF scaffolds (a, a′) and partial degradation of the scaffold structure (arrow in (a)). CF‐FB scaffolds showed markedly reduced cell infiltration and largely preserved scaffold integrity (b, b′). (c) The percentage of hematoxylin positive area was calculated as follows: the hematoxylin‐positive area divided by the total scaffold region defined by the large red dashed outline in (a) and (b). (d–f) DAPI staining confirms widespread nuclear signals throughout CF scaffolds (d, d′), whereas CF‐FB scaffolds exhibited minimal nuclear presence within the scaffold region (e, e′). (f) Quantification of DAPI positive area within the scaffold region. The percentage of DAPI positive area was calculated as the area of DAPI positive area divided by the total scaffold region defined by the large yellow dashed outline in (d) and (e). Data are presented as median with interquartile range (IQR), with individual data points shown. Statistical comparison between CF (*n* = 6) and CF‐FB (*n* = 6) was performed using two‐tailed Mann–Whitney *U* test (**p < 0.005).

We next examined whether this reduction in cellular infiltration was accompanied by decreased early immune cell accumulation. Because the T‐cell deficient nude mice model retains innate immune responses, it allowed us to evaluate early immune response without the contribution of mature T‐cell responses.^26^ CD45 and F4/80 immunostaining was used to detect leukocytes and macrophages, respectively.^27^, ^28^ CD45‐positive and F4/80‐positive cells were detected throughout CF scaffolds (Figure 4a,d), whereas their presence was reduced in CF‐FB scaffolds (Figure 4b,e). Quantitative analysis revealed that both positive areas of CD45 and F4/80 were significantly lower in CF‐FB scaffolds than in CF scaffolds (Figure 4c,f). These results indicate that FB cell sheet encapsulation attenuates leukocytes and macrophages' infiltration into implanted scaffolds.

**FIGURE 4:**
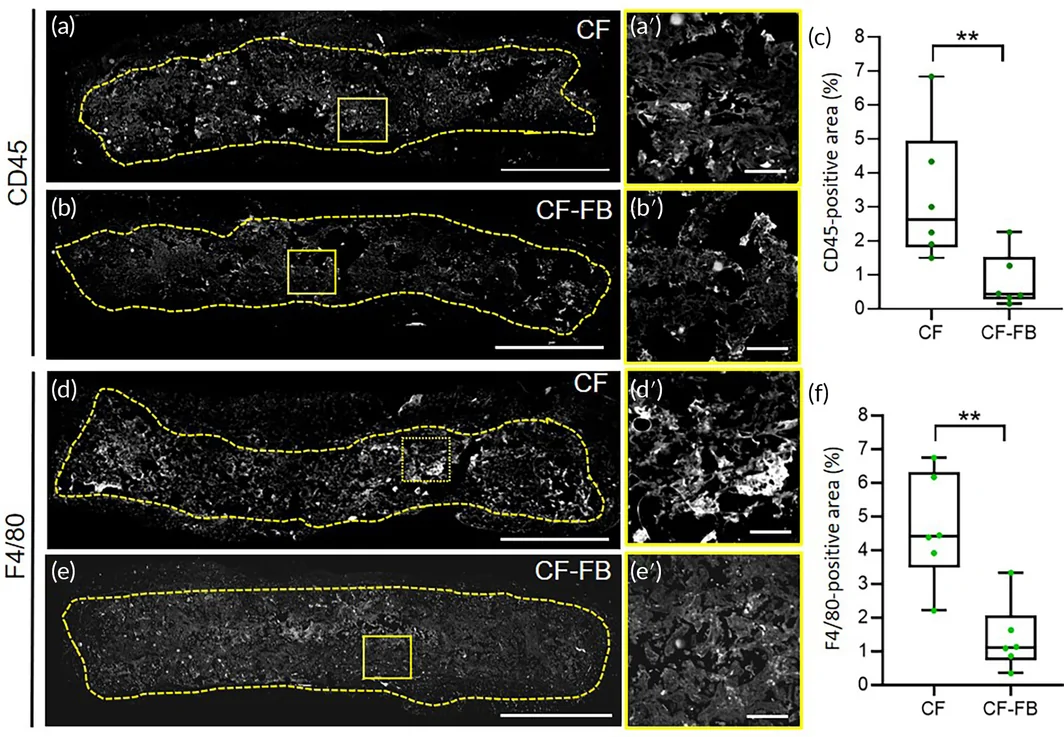
Fibroblast cell sheet wrapping attenuates early immune cell infiltration. (a–c) CD45‐positive cells were detected in CF scaffolds (a, a′), whereas fewer cells were observed in CF‐FB scaffolds (b, b′). (c) Quantification of CD45 positive area within the scaffold region. The percentage of CD45 positive area was calculated as the CD45 positive area divided by the total scaffold area defined by the large yellow outline (a, b). (d–f) Immunostaining for the macrophage marker F4/80 revealed F4/80‐positive cells in the CF scaffolds (d, d′), whereas fewer cells were observed in the CF‐FB scaffolds (e, e′). (f) Quantification of F4/80 positive area within the scaffold region. For both quantifications, data are presented as median with IQR, with individual data points shown. Statistical comparisons between CF (*n* = 6) and CF‐FB (*n* = 6) groups were performed using two‐tailed Mann–Whitney *U* test (**p < 0.005).

To further assess whether the encapsulating interface remained detectable after implantation, type I collagen (Col I) immunostaining was performed. In CF‐FB scaffolds, Col I signals were evident along the scaffold periphery, where they formed a discontinuous boundary‐like pattern (Figure S1, Supporting Information). In CF scaffolds, Col I staining was irregularly distributed throughout the explants and did not form a distinct peripheral boundary (Figure S1). This finding suggests that the cell sheet may function as a transient interface.

### 2.4 EPC incorporation restores cartilage ECM deposition

Having established the protective function of FB cell sheet encapsulation against host tissue infiltration, we next examined whether FB cell sheet encapsulation affected cartilage ECM formation within the scaffold. Safranin O/Fast green staining was used to assess cartilage ECM deposition, with cartilage matrix appearing pink to red and surrounding soft tissue appearing green to blue. Distinct Safranin O staining was evident throughout the CH scaffolds (Figure 5a), whereas the stained area was markedly reduced following FB cell sheet wrapping (Figure 5b). Notably, incorporation of EPCs into FB cell sheet (FB/EPC cell sheets) restored this stained area within the scaffold (Figure 5c). These observations were further supported by quantitative analysis (Figure 5d).

**FIGURE 5:**
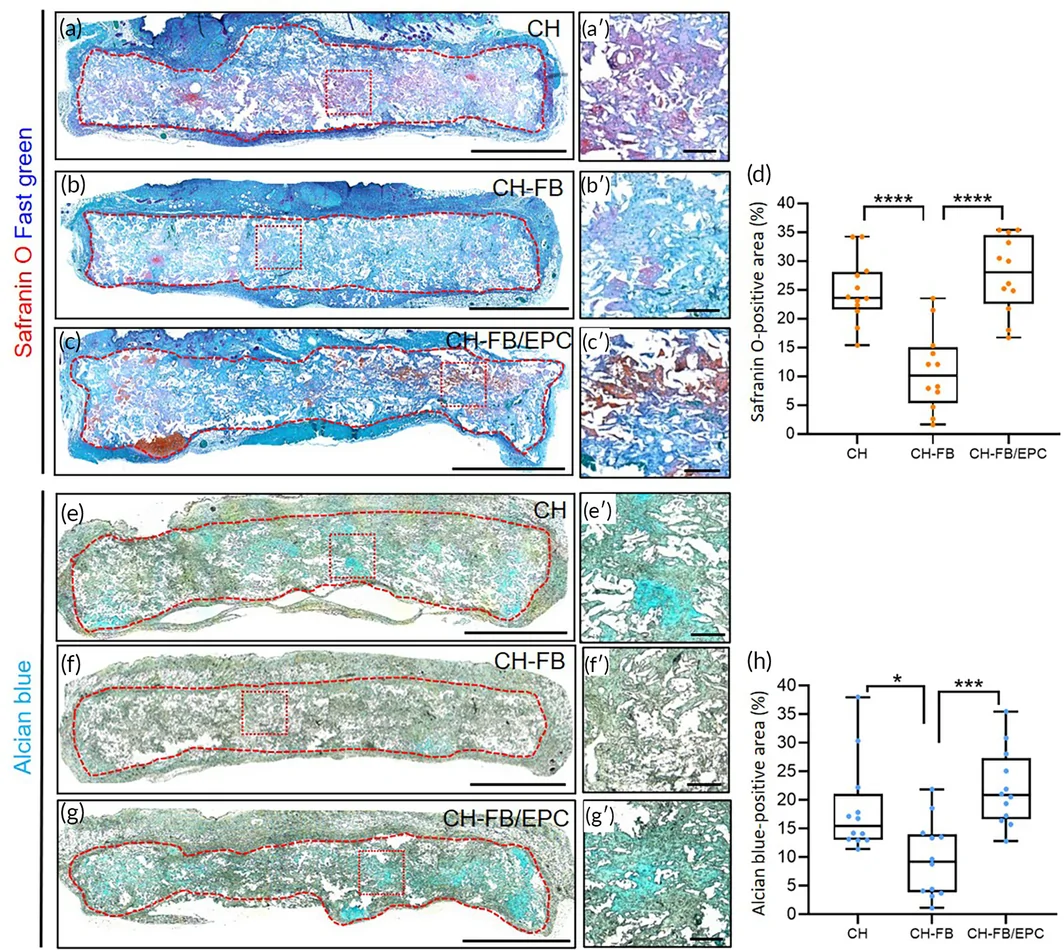
EPC incorporation restores cartilage ECM deposition. Chondrogenic matrix deposition was evaluated in chondrocyte‐laden scaffolds after 14 days of implantation. CH denotes unwrapped chondrocyte‐laden scaffolds. CH‐FB denoted scaffolds wrapped with a fibroblast cell sheet, and CH‐FB/EPC indicated scaffolds wrapped with EPC‐incorporated fibroblast cell sheet. Cross views were shown in the left panels (a–c, e–g), with boxed regions magnified in the right images (a′–c′, e′–g′). Scale bar = 2 mm (a–c, e–g), 200 μm (a′–c′, e′–g′). (a–d) Safranin O/Fast Green staining. Safranin O positive regions were observed throughout CH scaffolds (a, a′), whereas staining was markedly reduced in CH‐FB scaffolds (b, b′). Compared with the CH‐FB scaffold, the Safranin O^+^ regions were restored in CH‐FB/EPC scaffolds (c, c′). (d) Percentage of Safranin O positive area. (e–h) Alcian blue staining. Alcian blue signals were observed throughout the CH scaffolds (e, e′). In CH‐FB scaffolds, Alcian blue staining was markedly reduced (f, f′). In the CH‐FB/EPC group, Alcian blue staining was obviously increased compared with CH‐FB (f and f′, g and g′). (h) Percentage of Alcian blue positive area. Data are presented as median with IQR, with individual data points shown. Statistical comparisons between the scaffolds of CH (*n* = 12), CH‐FB (*n* = 12), and CH‐FB/EPC (*n* = 12) were performed using two‐tailed Mann–Whitney *U* test (*p < 0.05, ***p < 0.005, and ****p < 0.001).

Alcian blue staining for sulfated proteoglycan confirmed the distribution of cartilage ECM within the scaffold. Consistent with the results of Safranin O staining, distinct Alcian blue‐staining was observed throughout CH scaffolds (Figure 5e), whereas the stained area was markedly reduced following FB cell sheet wrapping (Figure 5f). In contrast, incorporation of EPCs restored Alcian blue‐positive matrix within the scaffold (Figure 5g). These differences were also reflected in the quantitative analysis of Alcian blue‐positive area (Figure 5h).

### 2.5 EPC incorporation promotes peri‐scaffold vascularization

Given the vascular potential of EPCs, we next examined the effect of EPC incorporation into the cell sheet on vascularization. Vascular structure was identified by CD31 immunostaining. CH‐FB/EPC scaffolds exhibited more prominent CD31 staining in the peri‐scaffold region (Figure 6c, ca), whereas only sparse and smaller CD31‐positive vascular structures were observed at the periphery of CH and CH‐FB scaffolds (Figure 6a and aa, b and ba). Quantitative analysis of CD31‐positive area further supported these observations, showing that the FB/EPC groups exhibited increased CD31‐positive area in the peri‐scaffold region compared with the other two groups (Figure 6d).

**FIGURE 6:**
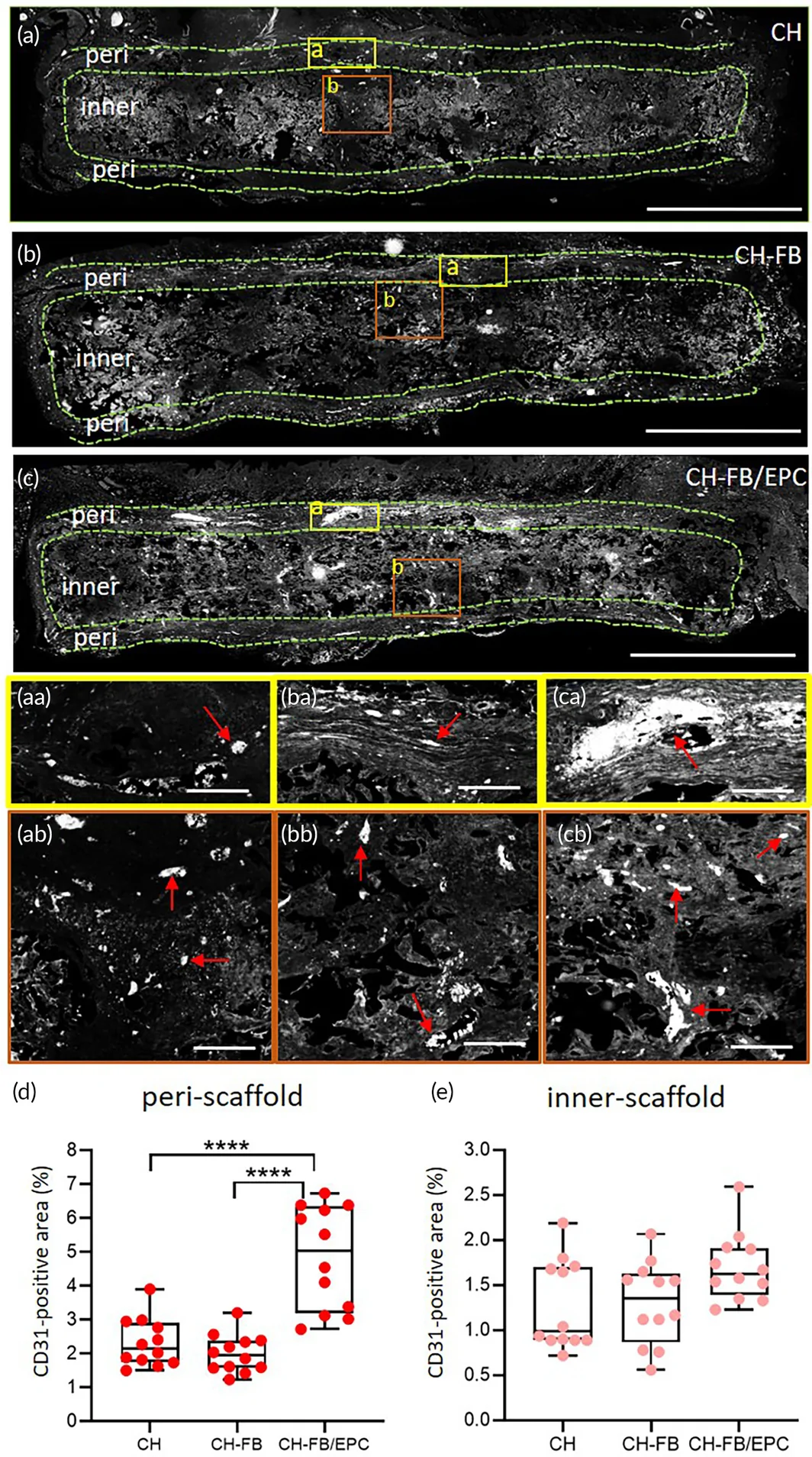
EPC incorporation promotes peri‐scaffold vascularization. (a–c) Representative CD31 immunostaining of CH (a), CH‐FB (b), and CH‐FB/EPC (c) scaffolds after 14 days of subcutaneous implantation. The green dashed outlines delineate the peri‐scaffold and inner scaffold regions used for regional quantification. The peri‐scaffold region was defined as the region approximately 200 μm adjacent to the scaffold boundary, while the remaining central region was defined as the inner region. The higher magnification images of the boxed peri‐scaffold regions and inner scaffold regions were shown in (aa–ca) and (ab–cb), respectively. Arrows indicate CD31‐positive structure. Scale bar = 2 mm (a–c), 200 μm (aa–ca, ab–cb). (d, e) Quantification of CD31 positive area in the peri‐scaffold (d) and inner (e) regions. The percentage of CD31 positive area was calculated as the CD31 positive area divided by the corresponding regional area defined by the green dashed outlines. Scaffolds of CH (*n* = 12), CH‐FB (*n* = 12 scaffolds), and CH‐FB/EPC (*n* = 12) were analyzed. Data are presented as median with IQR, with individual data points shown. Statistical comparisons were performed using two‐tailed Mann–Whitney *U* test (****p < 0.001).

In the inner scaffold region, no apparent differences in CD31‐positive staining were observed among these three groups (Figure 6a and ab, 6b and bb, 6c and cb). Consistently, quantitative analysis showed no statistically significant differences in CD31 positive area (Figure 6e). These results indicate that EPC incorporation enhanced vascularization predominantly at the scaffold periphery, without a significant increase within the scaffold interior.

### 2.6 Alternative encapsulation approaches exhibit distinct effects on scaffold protection and cartilage regeneration

Having established that cell sheet encapsulation reduces host cellular infiltration and supports cartilage regeneration, we investigated alternative encapsulation approaches to examine whether they could achieve similar effects. The polycarbonate (PC) membrane contains pores of approximately 0.4 μm, a configuration previously reported to prevent host cell infiltration while allowing nutrient diffusion.^29^ Thus, we examined the effects of PC membrane encapsulation on chondrocyte‐laden scaffold. CH scaffolds were encapsulated with the PC membrane seeded with FBs and EPCs. The PC membrane remained intact around the scaffold, with no apparent host cellular infiltration from the surrounding tissue into the scaffold (Figure 7a). Cartilage‐like regions characterized by lacuna‐like structures were observed only near the scaffold boundary (indicated by black dashed lines in Figure 7aa), whereas the scaffold interior contained sparse cellular structures and relatively limited tissue and matrix deposition (Figure 7ab). These results suggest that the PC membrane encapsulation limited host cellular infiltration but did not support cellular occupation and cartilage‐like tissue formation within the scaffold interior.

**FIGURE 7:**
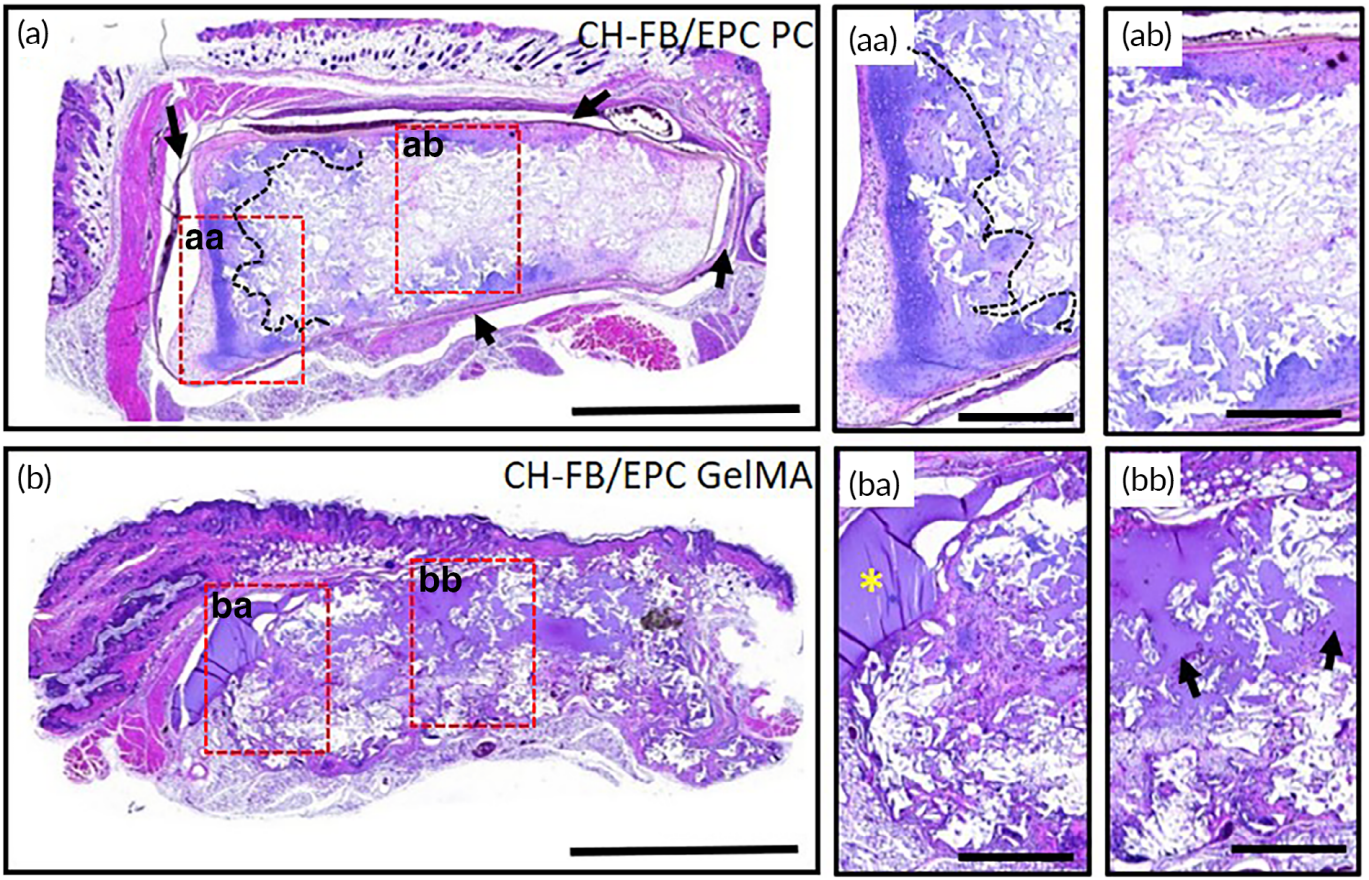
Different encapsulation strategies exhibit distinct effects on scaffold–host interaction and cartilage ECM deposition. (a) Representative H&E staining of CH‐laden scaffold encapsulated with a FB/EPC PC membrane (*n* = 4 scaffolds). Arrows in (a) indicate the PC membrane separating the CH scaffold and surrounding host tissue, with a distinct gap observed between the PC membrane and both scaffold and surrounding tissue. The box labeled “aa” in (a) indicates the scaffold peripheral region, shown at higher magnification in (aa), with cartilage‐like tissue with lacuna‐like structures near the scaffold boundary, delineated by a dashed line. The box labeled “ab” in (a) indicates the scaffold interior, shown in (ab), with a relatively pale H&E staining pattern and sparse cellular structures. (b) Representative H&E staining of CH‐laden scaffold encapsulated with a FB/EPC GelMA encapsulation (*n* = 4 scaffolds). The GelMA surrounding the scaffold was largely degraded, with only limited residual hydrogel remaining (ba). Asterisk indicates residual GelMA hydrogel around the scaffold (ba), while arrows in (bb) are GelMA remnants in the scaffold interior. Scale bar = 2 mm (a, b), 200 μm (aa, ab, ba, bb).

We next evaluated a GelMA‐based encapsulation approach, as GelMA has previously been used for encapsulating material for cartilage tissue‐engineered constructs.^30^ A thin layer of GelMA containing FBs and EPCs was applied to the surface of the CH scaffolds. After 14 days of implantation, only limited residual GelMA remained around the scaffold (asterisks in Figure 7ba), while the hydrogel remnants were observed in the scaffold interior (arrows in Figure 7bb). H&E staining revealed extensive and heterogeneous cellular infiltration throughout the scaffold (Figure 7ba, bb). Despite the extensive cellular presence, no apparent lacuna‐like structures were observed (Figure 7b). These results suggest that the GelMA layer underwent marked degradation after implantation, accompanied by limited retention of the encapsulating hydrogel and extensive cellular infiltration into the scaffold. No apparent cartilage‐like tissue formation was observed within the scaffold.

## 3 DISCUSSION

In this study, we developed a fibroblast cell sheet to encapsulate the PCL scaffold, providing a protective function that limits excessive host cellular infiltration and maintains scaffold integrity in a heterogeneous implantation environment. However, FB cell sheet encapsulation was also accompanied by reduced chondrogenic matrix formation within the scaffolds. Incorporation of endothelial progenitor cells (EPCs) into the cell sheet restored cartilage ECM deposition and enhanced vascularization around the scaffold. These results suggest that the engineered cell sheet can protect the scaffold from host tissue infiltration while functioning as a bioactive interface that modulates vascularization and supports cartilage regeneration.

Previous studies using synthetic membranes have reported the formation of a separating interface between scaffold and surrounding tissue.^12^, ^13^ In this study, the FB cell sheet formed a cohesive interface that directly contacts both scaffold and adjacent tissue while limiting excessive cellular infiltration (Figure 3). This difference suggests that the FB cell sheet can provide a protective interface while maintaining a more integrated cellular boundary with surrounding tissue. However, the protective effect was accompanied by reduced cartilage matrix formation within the scaffold (Figure 5). Previous studies have shown that cartilage constructs encapsulated by semipermeable membrane can maintain tissue formation, potentially because oxygen and nutrients can diffuse through the membrane.^12^, ^13^ In our study, the reduced matrix deposition following FB cell sheet encapsulation raises the possibility that the cell sheet barrier may have restricted oxygen and nutrient transport within the scaffold, thereby contributing to reduced chondrogenic matrix formation. Similar diffusion‐related limitations have been reported in other engineered tissue contexts. In islet transplantation, semipermeable encapsulation can protect grafts from immune‐mediated injury, but insufficient transport of oxygen and nutrients can limit graft survival and function.^14^, ^15^, ^16^ Likewise, in myocardial tissue engineering, stacking multiple cell sheets can result in hypoxia and functional impairment in the inner cell layer when adequate vascular integration is not established.^31^, ^32^ Overall, these findings suggest that although the FB cell sheet provides a cohesive and protective interface, this barrier may introduce a diffusion‐related limitation that constrains chondrogenic matrix formation within the scaffold.

To address this potential diffusion limitation, we incorporated EPCs into the cell sheet to introduce vascular potential. EPC incorporation preferentially enhanced peri‐scaffold vascularization, without significantly increasing vascularization within the scaffold interior. Vascularized tissue surrounding avascular cartilage, such as the perichondrium and subchondral bone, is known to contribute to cartilage homeostasis by providing nutrients and signaling molecules.^33^, ^34^ Similarly, vascular‐associated signals have been reported to support cell survival and chondrogenesis.^35^, ^36^, ^37^ Thus, the enhanced peri‐scaffold vascularization observed following EPC incorporation may establish a localized vascular source around the scaffold and potentially facilitate oxygen and nutrient transport. However, the contribution of peripheral vascularization to cartilage formation within engineered scaffolds remains poorly understood.

Although GelMA hydrogel underwent substantial degradation during subcutaneous implantation and was insufficient to maintain a stable protective layer under the subcutaneous environment, it remains an attractive material for hydrogel‐based encapsulation because it can provide an interface while permitting molecular diffusion and supporting a cell‐permissive microenvironment.^38^, ^39^ Therefore, incorporating complementary materials into GelMA may improve the stability and retention of the encapsulation layer after implantation. For example, PCL/GelMA composite systems have been reported to improve the persistence of GelMA‐based construct and slow their degradation than GelMA alone.^40^, ^41^, ^42^ Combining PCL/GelMA with EPCs may therefore provide a more durable encapsulating layer while retaining molecular transport and supporting EPC‐mediated vascularization. Other hydrogel formulations with improved persistence and cell‐supportive properties may also be explored as alternative encapsulation approaches, particularly those that can maintain a stable interface layer that limits host tissue infiltration while permitting molecular transport.

In this study, the salt‐leached PCL scaffold was further modified with laser‐generated macropores to facilitate chondrocyte infiltration into the scaffold interior, allowing evaluation of cartilage‐like tissue formation throughout the scaffold. Further fabrication approaches, such as 3D printing, could optimize the scaffold architecture by providing precise and reproducible control over pore size, porosity, and interconnectivity. In addition to 3D printing, electrospinning could provide a fibrous microarchitecture that more closely mimics the cartilage matrix and may support cell attachment and ECM deposition.^43^, ^44^ Therefore, both 3D printing and electrospinning represent promising approaches that could be integrated with the cell sheet‐based encapsulation strategy to further improve the performance of cartilage scaffolds after implantation.

Here, we used a subcutaneous implantation model to evaluate the interaction between the encapsulated scaffold and the surrounding host tissue. However, this model does not fully recapitulate the dynamic mechanical and tissue‐specific environment of orthotopic sites such as trachea and auricle. Therefore, future studies should evaluate the cell sheet encapsulation strategy in orthotopic transplantation models, such as tracheal scaffold transplantation or auricular cartilage implantation, to determine its performance within the native tissue‐specific environment and under the associated physiological mechanical loading, including multidirectional and cyclic mechanical forces.

Another limitation of this study is that male nude mice were used. Therefore, the observed host‐scaffold interactions and tissue responses should be interpreted within the context of male animals, and potential sex‐dependent differences cannot be excluded. Future studies including both sexes will be important to evaluate the universality and translational relevance of this strategy.

## 4 MATERIALS AND METHODS

### 4.1 Cell isolation and primary culture

Chondrocytes were isolated from New Zealand rabbit auricular cartilage. Cartilage tissue was minced into small fragments and digested with 0.1% collagenase at 37°C for 3 h. The digested solution was filtered through a 70‐μm mesh, centrifuged, and the collected cells were then cultured in DMEM (Gibco) supplemented with 10% FBS (Gibco) and 1× antimycotic (Thermo Fisher). Chondrocytes were sub‐cultured using TrypLE (Gibco).

Cells from the tracheal fibrovascular compartment were isolated from approximately 3 cm of trachea from New Zealand rabbits. After mechanically removing the luminal epithelial layer, the remaining tracheal tissue was digested with 0.1% collagenase for 1 h to minimize the release and mixing of chondrocytes. The digested solution was filtered with a 70‐μm mesh and then centrifuged. The collected cells were cultured in DMEM supplemented with 10% FBS and 1× antimycotic. These cells were sub‐cultured using TrypLE.

### 4.2 Isolation and enrichment EPCs

Endothelial progenitor cells (EPCs) were isolated from white adipose tissue in the groin region from New Zealand rabbit.^25^, ^45^ White adipose tissue was minced into small pieces and digested with 0.1% collagenase for 3 h at 37°C. After filtrating (70 μm mesh) and centrifuging the digested solution, the cells were treated with ACK lysis buffer to remove red blood cells. The resulting cells constituted the adipose‐derived stromal vascular fraction (SVF). SVF cells were seeded onto FNC (AthenaES)‐coated culture dishes and cultured in EGM‐2 medium (Lonza). Different sensitivity to TrypLE was used to enrich EPCs. Subculture of EPCs was performed using trypsin–EDTA (Gibco), and seeded in the EGM‐2 medium added ROCK inhibitor Y‐27632 (STEMCELL) to enhance cell survival.

### 4.3 Preparation of LbL‐assembled cell sheets

Cell sheets were fabricated using a layer‐by‐layer (LbL) assembly approach.^23^, ^24^ Tracheal fibroblasts at passage 3 were harvested using trypLE and resuspended with fibronectin coating mix (FNC) followed by 0.2% gelatin, and this coating cycle was repeated four times. Prior to seeding, cells were further coated with FNC. The coated cells were seeded onto 6‐well plates at 3.75 × 10^6^ cells per layer. Coated cells were seeded daily for four consecutive days. Two types of cell sheets were prepared: one was a four‐layer fibroblast cell sheet (FB cell sheet), and the other consisting of three fibroblast layers with a fourth layer of a mixture of EPCs and fibroblast (1:5) (FB/EPC cell sheet). Three days after the last cell seeding, the cell sheets formed a cohesive multilayered structure that could be completely peeled off from the culture dish.

### 4.4 Preparation of chondrocyte‐laden PCL scaffold

Poly(ε‐caprolactone) (PCL) scaffolds were fabricated using a salt‐leaching method as previously described.^20^, ^46^ PCL pellets (Sigma) were melted at 65°C and thoroughly mixed with sodium chloride (NaCl) particles. The mixture was then cast into planar molds (1 cm × 0.5 cm × 0.2 cm) and allowed to cool to room temperature to solidify. Subsequently, the NaCl particles were removed by immersion in deionized water. The scaffolds were further introduced with macropores using an UltraPulse® laser system. Laser ablation was performed at an energy of 20 mJ and a repetition rate of 300 Hz to generate through‐thickness macropores on the PCL scaffolds. PCL scaffolds were sterilized prior to cell seeding.

Chondrocytes at passage 3 were harvested and re‐suspended in 1× PBS at a density of 0.5 × 10^6^ cells per 50 μL. The cell solution was dropped onto both sides of the scaffolds, with a 1‐h interval between applications. After completion of cell seeding, the chondrocyte‐scaffolds were further incubated for 1.5 h, and then DMEM supplemented with 10% FBS and 1× antimycotic was added. After 3 days of culture, the medium was replaced with chondrogenic differentiation medium (DMEM with 50 μg/mL L‐ascorbic acid, 40 μg/mL L‐proline, 1.25 mg/mL BSA, 100 nM dexamethasone, 1× ITS, 10 ng/mL TGF‐β, and 5 ng/mL FGF‐2).

### 4.5 Whole mount immunofluorescent staining

Whole‐mount immunofluorescence staining was performed on chondrocyte‐laden scaffolds and engineered cell sheets. Samples were fixed in 10% formalin for 30 min, washed with 1× PBS, and blocked with 3% bovine serum albumin (BSA) in 1× PBS. Primary antibodies diluted in 1% BSA were applied overnight, followed by washing with 1× PBS and incubation with appropriate secondary antibodies for 1.5 h at room temperature. Antibodies used in whole mount staining were rabbit anti‐Fibronectin (FN) (ab11333), mouse anti‐CD31 (Novus, JC/70A), rabbit anti‐CD34 (ab81289), mouse anti‐Sox9 (ab76997), mouse anti‐aggrecan (ACN) (ab3773), mouse anti‐type II collagen (Col II) (ab185430). Secondary antibodies conjugated with Alexa Fluor® 488 (Abcam) were used, followed by DAPI and/or phalloidin‐594 (Thermo Fisher) counterstaining. Fluorescence images were acquired using a Leica confocal microscope, with z‐stack images collected for three‐dimensional reconstruction and analysis using LAS X software.

### 4.6 Experimental design

A total of 18 male nude mice (8 weeks old, weighing 20–25 g) were used in this study. Four subcutaneous pockets were created on the dorsal region of each mouse, and one scaffold was implanted into each pocket. The locations of four implantation pockets and the corresponding scaffold allocation are illustrated in Figure S2. The implanted groups consisted of cell free scaffold (CF, 8 scaffolds), cell‐free scaffold with FB cell sheet (CF‐FB, 8 scaffolds), chondrocyte‐laden scaffold (CH, 16 scaffolds), chondrocyte‐laden scaffold with FB cell sheet (CH‐FB, 16 scaffolds), chondrocyte‐laden scaffold with FB/EPC cell sheet (CH‐FB/EPC, 16 scaffolds), chondrocyte‐laden scaffold with FB/EPC PC membrane (CH‐FB/EPC PC, 4 scaffolds), and chondrocyte‐laden scaffold with FB/EPC GelMA (CH‐FB/EPC GelMA, 4 scaffolds).

Because the scaffold types were assigned to predefined and fixed implantation sites in each mouse as shown in Figure S2, randomization of scaffold allocation to implantation sites was not performed. Only male mice were included to maintain a consistent experimental background; therefore, potential sex‐dependent differences were not experimentally evaluated in this study.

### 4.7 Subcutaneous implantation

Male *CAnN.Cg‐Foxn1* ^*nu*^ */CrlNarl* nude mice were purchased from the National Laboratory Animal Center, Taiwan. All animal procedures were conducted in accordance with the Guide for the Care and Use of Laboratory Animals and were approved by the Institutional Animal Care and Use Committee (IACUC) of Chang Gung Memorial Hospital, Linkou, Taiwan (Protocol No. 2024011701). Mice were anesthetized with inhalational isoflurane (5% for induction and 2% for maintenance) during surgery. Under anesthesia, four separate skin incisions were made on the dorsal region of each mouse to create four independent subcutaneous pockets. A scaffold was implanted into each pocket, and the skin incisions were closed with 6–0 nylon sutures. After surgery, mice received gentamicin and ketoprofen once daily for three consecutive days and were monitored daily. At 14 days after implantation, mice were deeply anesthetized with isoflurane and subsequently euthanized by with Zoletil and Rompun. The implanted scaffolds with surrounding tissue were harvested.

### 4.8 Histological and immunofluorescence staining of tissue sections

For tissue analysis, explanted samples were fixed, paraffin‐embedded, sectioned, and deparaffinized. Hematoxylin and eosin (H&E), Safranin O, and Alcian Blue staining were performed according to standard histological procedures. For immunofluorescence staining, deparaffinized sections were followed by heat‐induced epitope retrieval (HIER) using Dako Target Retrieval Solution (Agilent) at 95°C for 20 min and cooled to room temperature. Sections were then blocked with 3% BSA for 1 h and incubated with primary antibodies at 4°C overnight. Primary antibodies used in tissue section staining included rabbit anti‐type I collagen (Col I) (ab34710), rabbit anti‐CD45 (ab10558), rabbit anti‐F4/80 (ab300421), and rabbit anti‐CD31 (ab182981). Secondary antibodies conjugated with Alexa Fluor® 488 were used, followed by DAPI counterstaining. Zeiss fluorescence microscope imaging and image capture were performed with AxioVision software.

### 4.9 Image quantification

Histological and immunofluorescence images were analyzed using ImageJ Fiji software (NIH). For H&E and Alcian blue staining, color deconvolution was processed using the built‐in hematoxylin and Alcian blue staining vectors, respectively.^47^ For Safranin O‐Fast green staining, stain vector was generated from representative regions using “From ROIs” function of the color deconvolution 2.^48^ For immunofluorescence images, individual channels were separated using “split channel” function, and the channels of interest were used for analysis. Quantification was performed using a fixed threshold and particle analysis applied to each staining experiment.

### 4.10 Statistical analysis

Samples that could not be analyzed because of slide preparation failure or unexpected animal death were excluded from the corresponding analysis as shown in Figure S2. The remaining evaluable samples were included in statistical analysis. Statistical analysis was performed using GraphPad Prism (version 11). Data were presented as median with interquartile range (IQR). The sample size (*n*) represented the number of evaluable implanted scaffolds for each group: CF (*n* = 6), CF‐FB (*n* = 6), CH (*n* = 12), CH‐FB (*n* = 12), and CH‐FB/EPC (*n* = 12). Differences between groups were examined using the two‐tailed Mann–Whitney *U* test. A *p*‐value <0.05 was considered statistically significant.

Post hoc power analysis was performed using G*power (version 3.1.9.7) based on the observed effect sizes of hematoxylin‐positive analysis area (%) and Safranin O‐positive area (%), and the actual numbers of evaluable scaffolds included in the corresponding statistical analyses, with a two‐tailed significance level of *α* = 0.05.

### 4.11 Encapsulation using polycarbonate membrane and GelMA hydrogel

A cellularized polycarbonate (PC) membrane was prepared by seeding 3.75 × 10^6^ cells onto each surface of a membrane with a total area of 9.6 cm^2^ (corresponding to a 3.5 cm dish). The membrane was used to wrap CH scaffold, with the FB layer facing the scaffold and the FB/EPC layer (5:1) facing outward. For hydrogel encapsulation, a GelMA solution (10% *w/v) containing FBs and EPCs (5:1, total cell density: 2 × 10^7^ cells/mL) was prepared with photoinitiator (0.1% Irgacure‐2959). GelMA solution was carefully dropwise applied onto the CH scaffolds to form a thin coating over the scaffold surface, and immediately polymerized under UV light (365 nm) for 5 min.

## AUTHOR CONTRIBUTIONS

**Yi Chieh Chang:** Investigation; writing – original draft; methodology; validation; visualization; formal analysis; data curation. **Cherng‐Shyang Chang:** Conceptualization; investigation; writing – review and editing; methodology. **Chung‐Kan Tsao:** Conceptualization; investigation; funding acquisition; writing – review and editing; project administration; supervision; resources; methodology.

## FUNDING INFORMATION

This work was supported by Chang Gung Memorial Hospital (CMRPG3N0121, CMRPG3N0122, and BMRPB03) and the National Science and Technology Council (NSTC) of Taiwan (112‐2314‐B‐182‐016, 113‐2314‐B‐182‐023‐MY3, and 114‐2811‐B‐182‐045).

## CONFLICT OF INTEREST STATEMENT

The authors declare no conflicts of interest.

## Supporting information


**Figure S1.** Partial retention of FB cell sheet at the scaffold periphery after implantation. (A) There was no distinct Col I‐positive boundary signal observed in CF scaffolds (A, Aa–Ac).
(B) Type I Collagen (Col I) immunostaining revealed Col I‐positive boundaries in selected regions around CF‐FB scaffolds (Ba, Bb), whereas Col I signal was less distinct in the scaffold margin shown in Bc. Scale bar = 2 mm (a, b), 200 μm (Aa–Ac, Ba–Bc).

**Figure S2.** Schematic representation of scaffold implantation sites and group allocation in nude mice. Representative dorsal view of a nude mouse showing the four scaffold implantation sites. One scaffold was implanted into each four subcutaneous pockets on the dorsal region. The red circles in left image indicated the locations of the implanted scaffolds. The left panel shows the allocation of scaffold conditions to the four implantation sites in each mouse. Excluded samples are indicated in the table, including slide preparation failure or unexpected animal death.

## Data Availability

The data supporting of the findings of this study are available from the corresponding author upon reasonable request.
